# ​​​​​Empowering Residents Through the Kuwait Family Medicine Review Course

**DOI:** 10.7759/cureus.86040

**Published:** 2025-06-15

**Authors:** Lulua Alasousi, Sara Alabdulhadi, Sara Alhammouri

**Affiliations:** 1 Family Medicine, Kuwait Institute for Medical Specializations, Kuwait, KWT; 2 General Practice, Kuwait Institute for Medical Specializations, Kuwait, KWT

**Keywords:** empowerement, family medicine residency, innovative teaching learning, innovative teaching strategies, residents under supervision of specialists (rss)

## Abstract

Empowerment plays a crucial role in advancing innovation within medical education. Research demonstrates that fostering autonomy and leadership among residents in medical residency programs significantly benefits both trainees and the healthcare system. The Kuwait Family Medicine Review Course has emerged as a pioneering initiative that has transformed the Kuwait Family Medicine Residency Program (KFMRP), serving as a model for educational advancement.

This paper examines the role of empowerment in medical education, with a particular focus on the Kuwait Family Medicine Review Course. It explores how resident-led teaching, leadership development, and academic collaboration contribute to enhancing residency training and strengthening Kuwait’s healthcare infrastructure.

Resident empowerment fosters leadership, autonomy, and innovation within medical education. The Kuwait Family Medicine Review Course exemplifies how structured initiatives can improve residency training, ultimately benefiting healthcare systems through better-prepared physicians. This model underscores the importance of empowerment-driven approaches in shaping the future of medical education.

## Editorial

Introduction

Resident empowerment in medical education is a crucial driver of professional development, skill acquisition, and innovation within healthcare systems [1]. Traditionally, residency training follows a structured mentorship model that limits residents' involvement in teaching and extracurricular activities. However, initiatives incorporating resident-led education, leadership roles, and autonomous decision-making have demonstrated significant benefits for both individual trainees and the broader healthcare community.

A notable example of such an initiative is the Kuwait Family Medicine Review Course, launched in 2016 by third-year family medicine residents - Drs. Sara Alhammouri, Sara Alabdulhadi, and Lulua Alasousi - under the supervision of the Kuwait Family Medicine Board [2]. Designed to prepare family medicine residents for careers as general practitioners, the course offers comprehensive updates on general practice, critical appraisal sessions, and hands-on clinical skills workshops. By fostering peer-led learning and academic collaboration, this initiative has contributed to advancing medical education and professional development within Kuwait's healthcare sector.

Resident-led learning: a transformative model

A defining feature of the Kuwait Family Medicine Review Course is its peer-led approach. For the first time in Kuwait’s medical education history, family medicine residents were empowered to deliver lectures to their peers on essential clinical competencies required for board examinations. This model promotes active learning, academic collaboration, and leadership development. The success of the review course has inspired the creation of additional educational initiatives within the family medicine discipline, including the Kuwait International Family Medicine Conference [3], the Kuwait Primary Health Care Conference, and the Focus Review Course, designed to equip residents for final board examinations.

Empowerment through leadership and autonomy

The Kuwait Family Medicine Review Course exemplifies how resident empowerment enhances job satisfaction, reduces burnout, and promotes innovation within residency programs [4]. Assigning residents key roles in organizing, teaching, and managing educational initiatives strengthens their sense of purpose and professional identity. In addition, residents develop essential fundraising, leadership, and decision-making skills that prepare them for future roles as family physicians, medical educators, and community healthcare leaders.

Senior residents contribute to the course by conducting workshops and lectures, including preparation sessions for the Membership of the Royal College of General Practitioners (MRCGP) Objective Structured Clinical Examination (OSCE). This structured mentorship model fosters collaborative learning environments, allowing residents to refine their teaching, leadership, and interpersonal communication skills.

Strategic planning and financial management

Beyond academic instruction, securing sponsorships from key stakeholders, including the Kuwait Ministry of Health, the Primary Care Medical Association, and pharmaceutical companies, is a fundamental component of the review course. This process necessitates event planning, budgeting, and resource management, all of which contribute to expanding professional networks and career prospects for participating residents.

Moreover, planning and executing a review course strengthens residents' strategic decision-making skills by requiring them to navigate complex logistical and financial challenges. Residents must allocate funds effectively, address unforeseen setbacks such as speaker cancellations, and ensure that educational initiatives run smoothly.

Fostering a culture of lifelong learning

Empowering residents through leadership roles fosters resilience and cultivates a commitment to continuous learning - a vital attribute in the evolving landscape of medical practice. By enabling residents to take ownership of their professional growth and teaching methodologies, the Kuwait Family Medicine Review Course establishes a supportive learning environment that prioritizes autonomy, collaboration, and innovation.

Institutional support and future implications

Under the leadership of Dr. Dina AlDubaib, the Kuwait Family Medicine Board continues to advocate for resident-led educational initiatives. The success of the Kuwait Family Medicine Review Course underscores the transformative impact of resident empowerment, setting a benchmark for medical education advancements and contributing to the enhancement of healthcare across Kuwait.

Conclusion

The Kuwait Family Medicine Review Course serves as a model of innovation within medical residency programs, demonstrating how resident empowerment fosters leadership, collaboration, and academic excellence. By equipping family medicine residents with the necessary skills to navigate complex challenges, lead educational initiatives, and engage in strategic decision-making, the program contributes to the strengthening of Kuwait’s healthcare system. Future residency programs should incorporate similar empowerment-driven models to ensure continuous improvement in medical education and professional development.
